# Misfolded SOD1 Associated with Motor Neuron Mitochondria Alters Mitochondrial Shape and Distribution Prior to Clinical Onset

**DOI:** 10.1371/journal.pone.0022031

**Published:** 2011-07-11

**Authors:** Christine Vande Velde, Karli K. McDonald, Yasmin Boukhedimi, Melissa McAlonis-Downes, Christian S. Lobsiger, Samar Bel Hadj, Andre Zandona, Jean-Pierre Julien, Sameer B. Shah, Don W. Cleveland

**Affiliations:** 1 Centre d'excellence en neuromique de l'Université de Montréal (CENUM), Centre de recherche du Centre hospitalier de l'Université de Montréal (CRCHUM), and Département de Médecine, Université de Montréal, Montréal, Québec, Canada; 2 Ludwig Institute for Cancer Research and Departments of Neuroscience and Cellular and Molecular Medicine, University of California San Diego, La Jolla, California, United States of America; 3 Institut National de la Santé et de la Recherche Médicale, Unité Mixte de Recherche S975, Centre de Recherche de l'Institut du Cerveau et de la Moelle Épinière, Hôpital de la Salpêtrière, Paris, France; 4 Centre de recherche du Centre hospitalier de l'Université Laval (CHUL), Université Laval, Québec, Québec, Canada; 5 Fischell Department of Bioengineering, University of Maryland, College Park, Maryland, United States of America; Johns Hopkins, United States of America

## Abstract

Mutations in superoxide dismutase (SOD1) are causative for inherited amyotrophic lateral sclerosis. A proportion of SOD1 mutant protein is misfolded onto the cytoplasmic face of mitochondria in one or more spinal cord cell types. By construction of mice in which mitochondrially targeted enhanced green fluorescent protein is selectively expressed in motor neurons, we demonstrate that axonal mitochondria of motor neurons are primary *in vivo* targets for misfolded SOD1. Mutant SOD1 alters axonal mitochondrial morphology and distribution, with dismutase active SOD1 causing mitochondrial clustering at the proximal side of Schmidt-Lanterman incisures within motor axons and dismutase inactive SOD1 producing aberrantly elongated axonal mitochondria beginning pre-symptomatically and increasing in severity as disease progresses. Somal mitochondria are altered by mutant SOD1, with loss of the characteristic cylindrical, networked morphology and its replacement by a less elongated, more spherical shape. These data indicate that mutant SOD1 binding to mitochondria disrupts normal mitochondrial distribution and size homeostasis as early pathogenic features of SOD1 mutant-mediated ALS.

## Introduction

Amyotrophic lateral sclerosis (ALS) is a prominent adult-onset motor neuron disease characterized by the progressive and selective loss of motor neurons of the motor cortex, brainstem and spinal cord [1]. While the cause of the majority of cases remains unknown, ∼10% of instances are genetically inherited, with the most frequent cause being mutations in the ubiquitously expressed Cu,Zn superoxide dismutase 1 (SOD1). The nature of SOD1 mutant toxicity and its selectivity for motor neurons remains unsettled. Seven highly divergent cellular mechanisms for mutant SOD1-mediated damage have been proposed [2], including inhibition of the voltage dependent ion channel (VDAC1) [3] and protein import through the TOM complex [4] following binding of misfolded mutant SOD1 onto the cytoplasmic face of spinal cord mitochondria [5]. These latter interactions are unique to spinal mitochondrial membranes, but the cell type(s) in which this interaction(s) occurs is not established.

Disturbed mitochondrial ultrastructure in motor neurons and muscles in both sporadic and familial ALS first implicated mitochondria in ALS pathogenesis [6]–[9]. Similar alterations of mitochondria, including vacuolated, dilated and disorganized mitochondria were later identified in spinal motor neuron cell bodies of mutant SOD1 mouse models expressing dismutase active [10]–[13], but not inactive mutants [14]. The frequency of these abnormalities and their neuronal distribution, especially within axons, has not been determined. Through generation of a transgenic mouse line with mitochondrially targeted EGFP expressed only in motor neurons, we now identify abnormally swollen mitochondria within motor neuron cell bodies, reduced number of axonal mitochondria, and a highly surprising misdistribution along axons as common features of early pathogenesis in SOD1-mediated inherited ALS.

## Results

### A transgenic mouse line with selective fluorescent labeling of motor neuron mitochondria

**Figure 1:**
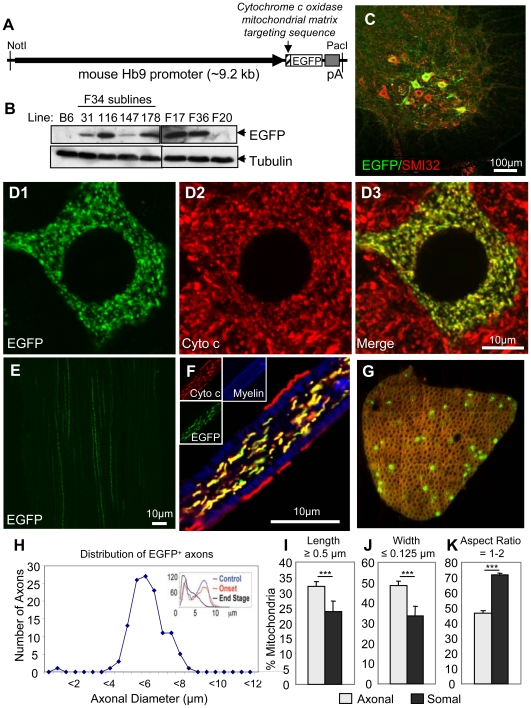
Generation of a novel transgenic mouse with mitochondria labeled uniquely in motor neurons. (A) Schematic of Hb9-MitoEGFP transgene. (B) Immunoblot of spinal cord homogenates of Hb9-MitoEGFP founders (F34, F17, F36, F20) and F34 sublines probed for EGFP and tubulin (loading control). (C–G) MitoEGFP *(green)* expression in spinal cord motor neurons (C & D), sciatic nerve (E), and L5 motor axons (F & G) labeled with SMI32 (C, *red*), cytochrome *c* (D & F, *red*), and Fluoromyelin Red (F & G, *blue/red*). Note that mitochondria have typical tubular and punctate morphologies and MitoEGFP expression is excluded from Schwann cells. (G & H) 15% of large caliber (>4.5 µm) L5 motor axons express MitoEGFP in 12 months animal. The expected biphasic distribution of axonal caliber in adult C57Bl/6 mice is indicated in the upper right corner. Comparison of axonal and somal mitochondrial length (I), width (J) and aspect ratio (K) in Hb9-MitoEGFP mice. Scale bars, 10 µm.

To assess the relative distributions and morphologies of mitochondria uniquely within motor neurons, four transgenic mouse lines were generated in which EGFP was selectively synthesized by motor neurons and directed into mitochondria (**Fig. 1A, B**) by in frame ligation to the 25 amino acid targeting sequence from the mitochondrial matrix component Cytochrome *c* oxidase subunit VIII. This fusion gene was placed under the transcriptional control of the mouse *Hb9* promoter, whose expression is unique to the motor neuron lineage [15], [16] and whose expression persists in 10–20% of adult motor neurons [17]. Immunofluorescence of spinal cord sections was used to determine that varying levels and patterns of MitoEGFP were present in the different lines, with the highest level and motor neuron-restricted expression found in subline 34–116. This line, referred to hereafter as Hb9-MitoEGFP, was maintained hemizygous and backcrossed three generations into C57Bl/6 to generate mice enriched in this background.

Hb9-MitoEGFP mice were phenotypically normal with typical gait, posture, and hind-limb reflexes. Consistent with the expected mitochondrial pattern, EGFP accumulated in a speckled pattern within motor neurons of the ventral spinal cord and was comprised of a mix of both tubular and punctate morphologies (**Fig. 1C, D**). Higher magnification images confirmed co-localization of MitoEGFP with the mitochondrial marker cytochrome c in nearly all mitochondria accumulating MitoEGFP (**Fig. 1D**). Occasional mitochondrial labeling was also observed in a few small neurons near the central spinal canal. These cells are likely to be interneurons and were excluded from subsequent analyses.

Mitochondria were detected in long linear tracts in axons within the sciatic nerves of Hb9-MitoEGFP mice (**Fig. 1E, F**). Within ventral roots, MitoEGFP was restricted to axonal mitochondria and excluded from myelinating Schwann cells (**Fig. 1F**). Fifteen percent of L5 ventral axons contained Hb9 driven MitoEGFP (**Fig. 1G**), all of which were the large-caliber (>4.5 µm; **Fig. 1H**) axons at-risk in ALS [18] and SOD1 mutant mediated ALS-like disease in mice [14]. Morphology of EGFP-marked mitochondria was indistinguishable from those in non-EGFP expressing neurons (**Fig. 1D**).

### Axonal and somal mitochondria are morphologically distinct

In both normal mitochondria and those expressing EGFP the morphology of mitochondria was different between the cell body and axonal compartments with both found in an interconnected network within motor neuron cell bodies, while axonal mitochondria were present as distinct entities often with an elongated appearance (compare **Fig. 1D, F**). Mitochondrial length, width, and aspect ratio (the ratio of length to width) within motor axons and somata of Hb9-MitoEGFP mice were determined (90% of individual morphologies were scored with semi-automated custom software, with the remaining 10% separated manually owing to more complex connecting linkages). A greater proportion of axonal mitochondria were longer than 0.5 µm compared with those in the soma (32.1±1.5% vs. 23.8±3.5%, p<0.05) (**Fig. 1I**). Furthermore, by 8 months of age, average mitochondrial length was increased by ∼50% in axons compared to cell bodies (0.91±0.03 µm vs. 0.64±0.01 µm, p<2×10^−40^). While mitochondrial diameters (i.e., width in one-dimension) have been reported in most cellular contexts as 200–300 nm [19], the population of axonal mitochondria were of significantly (p<0.03) smaller diameters (e.g., 48.9±1.9% of axonal mitochondria had diameters less than 125 nm, compared to 33.7±4.8% in the soma) (**Fig. 1J**). Indeed, somal mitochondrial diameters (widths) were 20% larger than axonal mitochondria (0.36±0.01 µm vs. 0.30±0.01 µm, p<1×10^−37^). Aspect ratio provided an unbiased measure of asymmetry in shape: the proportion of punctal mitochondria (aspect ratio near 1) within axons was markedly reduced compared to motor neuron cell bodies (46.4±1.7% vs. 71.6±1.4%, p<0.00002) (**Fig. 1K**). We conclude that there are significant compartmental differences in mitochondrial morphology between cell bodies and axons of motor neurons.

### Misfolded SOD1 associates with axonal mitochondria *in vivo*

We exploited the Hb9-MitoEGFP mouse to visualize mitochondrial distribution and morphology in the context of mutant SOD1-mediated ALS in previously characterized transgenic mouse lines expressing either of two ALS-linked SOD1 mutants, SOD1^G37R^ (dismutase active) and SOD1^G85R^ (dismutase inactive). These two mutant SOD1 lines develop clinical disease features of early paralysis and muscle wasting at 5 and 11 months, respectively. Introduction of the Hb9-MitoEGFP transgene did not significantly change the life span or phenotype of these animals. Using a monoclonal antibody (DSE2) raised against the carboxyl-terminal domain of SOD1 that is inaccessible in properly folded SOD1, immunoprecipitation has previously been used to demonstrate the preferential association of misfolded SOD1 with the surface of spinal cord mitochondria [5] and that a portion of this mitochondrially associated mutant SOD1 is bound directly to VDAC1 [3].

**Figure 2:**
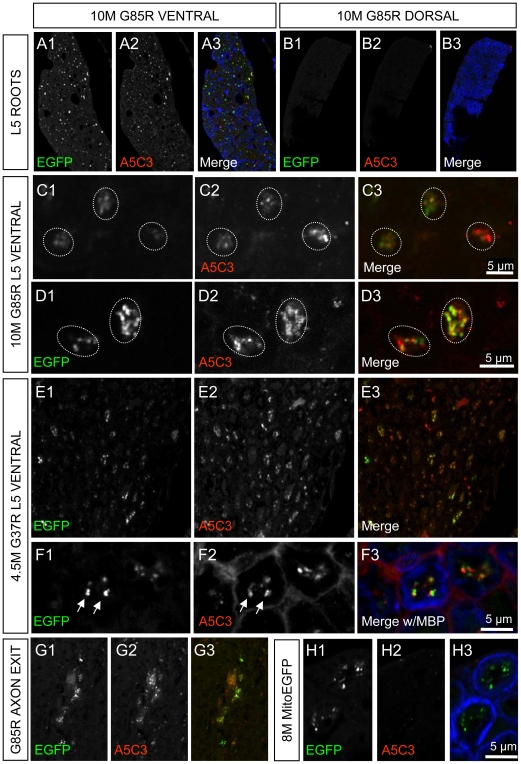
Misfolded SOD1 associates with motor neuron axonal mitochondria *in vivo*. An antibody raised against misfolded SOD1 (A5C3, *red*) labels ventral (A) but not dorsal (B) axons of 10 month SOD1^G85R^ mouse. Myelin Basic Protein (MBP, *blue*) is included as a counter-label. Misfolded SOD1 (A5C3, *red*) is often colocalized with mitochondria (EGFP, *green*) in motor axons of both SOD1^G85R^ (C & D) and SOD1^G37R^ animals (E & F) but not non-SOD1 MitoEGFP littermates (H). Regions of the spinal cord, consistent with axonal exit zones also demonstrate double-labeling (G). The boundaries of individual axons are indicated either by MBP labeling (*blue*) or dotted lines. Scale bars are as indicated.

To determine in which cell type(s) this misfolded mutant SOD1 accumulates, we took advantage of an additional monoclonal antibody (mAb A5C3) that is suitable for immunofluorescence and which uniquely recognizes a misfolded SOD1 epitope within exon 4 [20]. Examinations of spinal cord sections from early symptomatic (10 month) SOD1^G85R^ mice revealed an enrichment of misfolded SOD1 within motor neuron axons as visualized in L5 ventral roots (**Fig. 2A**). Misfolded SOD1 was completely absent in sensory axons of the dorsal root (**Fig. 2B**). Closer inspection of ventral axons revealed frequent colocalization of misfolded SOD1 with motor axonal mitochondria, as labeled with MitoEGFP, in both early symptomatic SOD1^G85R^ (**Fig. 2C, D**) and SOD1^G37R^ mice (**Fig. 2E, F**). To determine the extent of colocalization, we used non-biased imaging software to quantify the overlap coefficient between individual mitochondria labeled with MitoEGFP and mAb A5C3, where a value of one represents the maximum degree of colocalization. By this approach, we determined the average overlap coefficient between MitoEGFP and mAb A5C3 as 0.913±0.007 and 0.915±0.009 for SOD1^G85R^ and SOD1^G37R^ ventral axons, respectively. We also noted some enrichment of misfolded SOD1 which partially corresponded with mitochondrial labeling at sites consistent with axonal exit zones (**Fig. 2G**). Misfolded SOD1 was not detectable in the ventral axons of MitoEGFP mice under the same conditions, as expected (**Fig. 2H**). These data establish that misfolded SOD1 primarily accumulates on (and/or in) motor axon mitochondria *in vivo*.

### Mutant SOD1 changes to somal and axonal mitochondria in motor neurons

**Figure 3:**
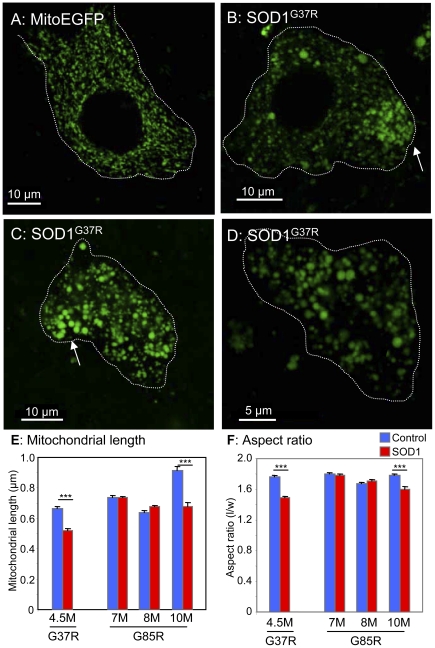
Motor neuron mitochondria are rounder in mutant SOD1 mice. MitoEGFP (*green*) expression in spinal cord motor neurons in control MitoEGFP (A) and SOD1^G37R^ early symptomatic animals (B–D). The control neuron has normal mitochondria of diverse shapes and sizes. In contrast, SOD1^G37R^ motor neurons have rounded swollen mitochondria with uneven distribution. Arrows indicate possible axon hillocks. Motor neuron boundaries have been outlined with dotted line. Quantification of mitochondrial length (E) and aspect ratio (F) in motor neuron cell bodies of mutant SOD1 animals of various ages and age-matched MitoEGFP control animals. ***, p<0.0005. Scale bars, 10 µm.

**Figure 4:**
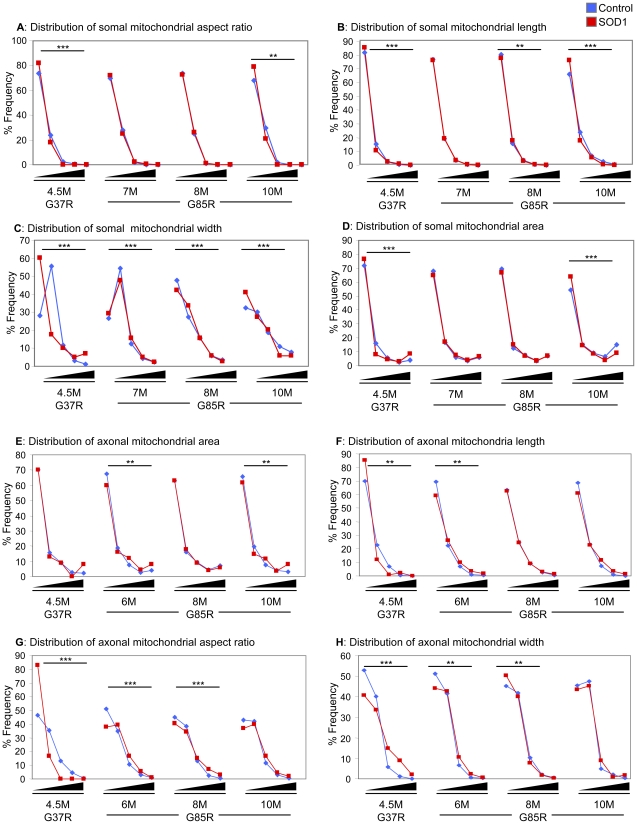
Distributions of mitochondrial morphology and distribution. Distributions in various morphological parameters of somal (A–D) and axonal (E–H) mitochondria. Bins for area (A,E) are 0–0.25, 0.26–0.50, 0.51–0.75, 0.76–1.0, and >1.0 µm^2^. Bins for length (B,F) are 0–1, 1.01–2, 2.01–3, 3.01–4, and >4 µm. Bins for aspect ratio (C,G) are 1–2, 2.01–4, 4.01–6, 6.01–8, and >8. Bins for width (D,H) are 0–0.25, 0.26–0.50, 0.51–0.75, 0.76–1.0, and >1.0 µm. Statistics have been calculated using Chi-square with Yates' correction for continuity and significant differences in distributions are indicated: *, p<0.05; **, p<0.005; ***, p<0.0005.

Age-dependent changes were seen in mitochondria of motor neurons within SOD1 mutant mice. By 4.5 months, the initial mitochondrial network within motor neuron perikarya of SOD1^G37R^ mice became less connected, with discrete, nearly circular (with aspect ratio near 1) mitochondria appearing more often (compare **Fig. 3A–D**). Many of these mitochondria had strikingly large (diameters >1 µm) (**Fig. 3B–D**), with average mitochondrial length and aspect ratio reduced 22% and 15%, respectively (**Fig. 3E, F**; **Fig. 4**). Similar reductions were identified in length (26%) and aspect ratio (10%) in 10 month (but not 7 or 8 month) SOD1^G85R^ animals. Rounding of motor neuron mitochondria in both SOD1^G85R^ and SOD1^G37R^ mice was achieved by a shortening of mitochondrial length (**Fig. 4B**), rather than increased width (**Fig. 4C**).

**Figure 5:**
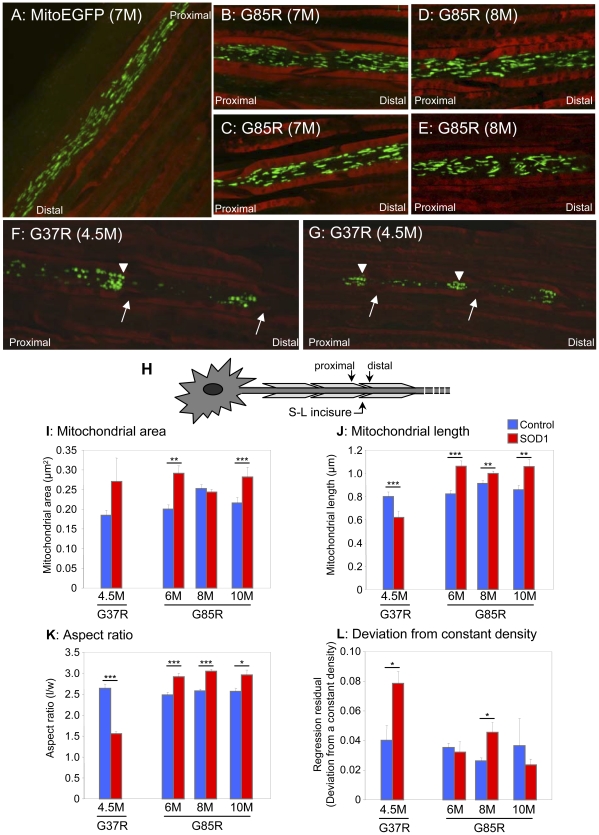
Mitochondria morphology is altered in mutant SOD1 axons. MitoEGFP (*green*) expressed in the sciatic nerves in control (A), asymptomatic SOD1^G85R^ 7 months (B and C), SOD1^G85R^ 8 months (D and E), and SOD1^G37R^ early symptomatic (F and G) labeled with Fluoromyelin red (*red*). Round and evenly distributed mitochondria are seen in both SOD1^G85R^ (symptomatic stage) and SOD1^G37R^ (early symptomatic stage). Mitochondria `pile-up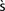 or clusters are also seen in the proximal side of SLIs (arrows) in the SOD1^G37R^ axons. Arrowheads indicate “strings” of mitochondria. Arrows indicate SLIs (H) Schematic indicating proximal and distal locations of SLIs. Mitochondrial area (I), length (J), aspect ratio (K), and density (L) were evaluated in motor axons of the sciatic nerves of and mutant SOD1 animals of various ages and age-matched MitoEGFP control animals, as described in detail in the Materials and Methods. Statistics are indicated: *, p<0.05; **, p<0.005; ***, p<0.0005.

In the absence of mutant SOD1, mitochondria (both tubular and punctal) of Hb9-MitoEGFP mice were distributed homogenously along the length of each motor axon (**Fig. 5A**). In contrast, in SOD1^G85R^ (**Fig. 5B–E**) and SOD1^G37R^ mice (**Fig. 5F, G**) mitochondria became progressively smaller, rounder, and unevenly distributed along axons. Strings of 3–4 mitochondria abutting each other much like pearls on a string were frequently found in axons from both SOD1 mutants (**Fig. 5E–G**), consistent with increased mitochondrial fission and/or decreased fusion. Mitochondrial area (calculated based on the number of pixels composing each separate object) within SOD1^G37R^ and SOD1^G85R^ axons and (**Fig. 5I**) increased 45% and 27% in axons of 6 and 10 month old SOD1^G85R^ animals, respectively (6 months: 0.29±0.02 vs. 0.20±0.01 µm^2^, p<0.00003; 10 months: 0.28±0.01 vs. 0.22±0.01 µm^2^, p<0.02). Similarly, a trend almost reaching statistical significance for increased (by 50%) mitochondrial area (p<0.08) was detected in early symptomatic SOD1^G37R^ axons (0.27±0.06 vs. 0.18±0.01 µm^2^).

In both mutant SOD1 lines, these increases reflect progressive increases in the areas of the largest mitochondria (right-most bin), at the expense of smaller mitochondria (**Fig. 4E**). Each mitochondrion was fit to an elliptical shape so as to define length and width for each mitochondrion (**Fig. 5J**). In SOD1^G37R^ animals, the observed changes in mitochondrial area were accompanied by a statistically significant, 22% decrease in length (**Fig. 5K**) and 48% increase in width (**Fig. 4H**), respectively. This was reflected in a significant 41% reduction in aspect ratio, indicating that SOD1^G37R^ mitochondria are becoming less tubular and increasingly round (1.56±0.05 vs. 2.64±0.10, p<4.2×10^−19^) compared to age-matched controls (**Fig. 4G**, **Fig. 5K**). In SOD1^G85R^ axons, at all time points examined, mitochondrial lengths were observed to be increased relative to age-matched controls (**Fig. 4F**, **Fig. 5J**), resulting in an average 17% increase in aspect ratio (2.97±0.04 vs. 2.54±0.03, p<0.0008) (**Fig. 5K**). Histograms confirmed that at very early, pre-symptomatic ages, SOD1^G85R^ axonal mitochondrial were longer (though not necessarily narrower), and thus more tubular compared to non-SOD1 transgenic controls (**Fig. 4E–H**).

### Mutant SOD1 induces misdistribution of mitochondria in motor axons

In the absence of mutant SOD1, axonal mitochondria distributions were relatively homogeneous throughout the length of the axons (**Fig. 5A**). Mitochondrial distribution in the motor axons in sciatic nerves of Hb9-MitoEGFP mice expressing either SOD1^G37R^ or SOD1^G85R^ mutations was markedly different. At the earliest phenotypic stages, mitochondrial distributions were obviously disrupted in SOD1^G37R^ animals (**Fig. 5F, G**), including reduced mitochondrial densities (measured as the number of mitochondria per axonal segment) in early symptomatic SOD1^G37R^ and SOD1^G85R^ animals. Mean residual regression analysis of mitochondrial distribution as a function of the deviation from a constant density confirmed increased variability in mitochondrial axonal distribution of early symptomatic SOD1^G37R^ and pre-symptomatic 8 month SOD1^G85R^ axons. The magnitude of residual regression was increased nearly 2-fold in SOD1^G37R^ motor axons (0.079±0.008 vs. 0.040±0.010, p<0.04) and 1.8-fold in 8 month SOD1^G85R^ axons (0.046±0.007 vs. 0.026±0.002, p<0.01) compared to respective age-matched controls (**Fig. 5L**). (Small values for regression residuals reflect a constant density along the axon, while larger values reflect variable mitochondrial density.).

The most striking feature was preferential accumulation of mitochondria within SOD1^G37R^ axons (**Fig. 5F, G**) just proximal to Schmidt-Lanterman incisures (SLI), readily identifiable structures in peripheral myelin where a small amount of Schwann cell cytoplasm has been displaced. These sites correspond to areas of increased neurofilament packing, thus restricting axonal diameter [21], [22]. The functions of SLIs are not established, but they are believed to participate in axon-glial communication [21], [22]. When the average mitochondrial density in all axonal regions distal to the incisures was compared to the density for a 7.0 µm region (two 3.5 µm segments/bins) proximal to each incisures, there was a striking 6-fold decrease in mitochondrial density at the distal side of incisures of SOD1^G37R^ axons (1.9±0.5 vs. 0.3±0.1, p<0.04). In contrast, no significant mitochondrial clustering at SLIs was detected in SOD1^G85R^ axons at any time point examined.

## Discussion

The maintenance of optimal mitochondrial function, transport and distribution is essential to motor neuron survival [23]. Using a new transgenic mouse model which permits the direct visualization of axonal and somal mitochondria of motor neurons, we report important differences in mitochondrial morphology in these two compartments (**Fig. 1**). Where prior efforts had identified preferential deposition of misfolded SOD1 onto some spinal cord mitochondria [5], [24], including portions directly complexed to VDAC1 [3] or the anti-apoptotic BCL-2 [25], or impacting the TOM mitochondrial protein import channel [4], we have now established that a component of disease common to dismutase active and inactive SOD1 mutants is misdistribution and altered morphology of axonal mitochondria within motor neurons accompanied by intra-axonal accumulation of misfolded SOD1. Mitochondria within motor neuron cell bodies are also noticeably swollen (shifting from tubular) and less interconnected morphologies (**Fig. 2**). Considering that axons account for 99% of the total motor neuron volume, and the relative abundance of mitochondria within these axons, the disturbances in axonal mitochondrial geometry and distribution we describe here would be expected to have a significant impact on the overall function of the motor neuron.

Perhaps even more striking is that our evidence has revealed the common finding that somal mitochondria become shorter and rounder in both dismutase active and inactive mutant SOD1 lines (**Fig. 3**). In the case of SOD1^G37R^, grossly enlarged mitochondria with diameters exceeding 1 µm were found. However, in axons, where well-organized and tightly packed cytoskeletal filaments may impose additional geometric restrictions on mitochondrial shape [26], we observed significant changes in mitochondrial morphology in these mutant SOD1 lines with SOD1^G37R^ mitochondria shifting from long, tubular mitochondria to punctate mitochondria, while SOD1^G85R^ mitochondria underwent an increase in length. These morphological changes are accompanied by the development of a non-homogenous mitochondrial distribution along the length of the axon, most striking of which are the focal concentrations of mitochondria within SOD1^G37R^ axons at the proximal sides of SLIs (**Fig. 5**). Our findings are consistent with mitochondrial morphology observed following acute injury (e.g., intercostal nerve-muscle explants) where long, tubular, extended mitochondrial forms shifted noticeably to smaller, immobile ones [27]. While both mutant SOD1s clearly alter axonal mitochondrial morphology and distribution, the final mitochondrial shape and positioning differs in axons of animals expressing each mutant. Since there are intrinsic differences in dismutase activity between these two mutants, this may account for the differences in mitochondrial morphology and distribution triggered by each mutant.

The morphological changes to mitochondria are accompanied by the development of a non-homogenous mitochondrial distribution along the length of motor axons, most striking of which are the focal concentrations of mitochondria within SOD1^G37R^ axons at the proximal sides of SLIs. From this, we propose that the clusters of mitochondria containing mutant SOD1 observed at regular intervals in axoplasm from teased nerve whole mounts prepared from pre-symptomatic rats expressing dismutase active SOD1^G93A^ [28] represent similar mitochondrial pileups at the proximal sides of SLIs.

The marked change in mitochondrial geometry is likely of functional significance since it has previously been demonstrated in cultured cerebellar granule neurons that agents which induce mitochondrial swelling without affecting ATP production cause a severe impairment in mitochondrial motility [19]. Thus, major alterations in mitochondrial geometry can impair trafficking, without affecting energy production. Similarly, altered mitochondrial morphology is observed following acute injury (e.g., in intercostal nerve-muscle explants where long, tubular, extended mitochondrial forms shifted to smaller, immobile grains [27]. That mitochondrial swelling may be a key component in ALS is further supported by a recent claim that deletion of Cyclophilin D, a regulator of the mitochondrial permeability transition pore, itself a regulator of mitochondrial volume, successfully reduced mitochondrial swelling and delayed disease in SOD1^G93A^ mice [29] (note, however, that our own unpublished efforts with a similar genetic approach do not confirm the reported benefit; S. Da Cruz, P. Parone, and D.W.C). Further, deletion or overexpression of VDAC1 in *Drosophila* indirect flight muscles results in elongated and fragmented mitochondria, respectively [30].

The pileup of mitochondria in dismutase active SOD1 mutants that we have documented at the proximal sides of the SLIs mirrors mitochondrial changes in morphology and depletion from axons following axotomy, in the latter case through a combination of decreased anterograde motor activity for mitochondria without a change in stationary phases or alteration in retrograde mitochondrial movement [31]. Indeed, enhanced retrograde movement of mitochondria, and consequent depletion of mitochondria at distal terminals in SOD1^G93A^ embryonic motor neurons or transfected cortical neurons has been linked to perturbation of the anterograde component of fast axonal transport and mutant SOD1 has been shown to slow anterograde transport within axoplasm *in vitro* [31], [32]. In light of this and our findings here using a novel transgenic mouse line to report uniquely on motor neuron mitochondrial distribution and geometry, we now propose that misfolded SOD1 within motor neurons inhibits mitochondrial ability to regulate their volume (and morphology), which in turn negatively impacts their axonal positioning, at least in part by affecting anterograde axonal transport within motor axons as an early, and possibly central aspect of disease pathogenesis. In addition, the development of this novel transgenic line Hb9-MitoEGFP has revealed an important impact of misfolded SOD1 on motor axonal mitochondria which was not previously appreciated (or possible to detect). Further investigation of this mechanism is likely to yield important therapeutic targets.

## Materials and Methods

### Ethics statement

Animals were treated in strict accordance with approved protocols from the Centre de Recherche du Centre Hospitalier de l'Université de Montréal (CRCHUM) Institutional Committee for the Protection of Animals (N08001CVsr) and the University of California, San Diego Institutional Animal Care and Use Committee (S00225). Both committees follow national standards as outlined by the Canadian Council on Animal Care (CCAC) and the American Association for Laboratory Animal Science (AALAS), respectively. All possible efforts were made to minimize any suffering.

### Generation of transgenic mice

The pHb9-MitoEGFP was generated by introducing the mitochondrial targeting sequence of Cytochrome *c* Oxidase subunit VIII into pHb9-EGFP via standard cloning techniques. Transgenic founders (from F1 C57Bl/6 parental mice) were generated by pronuclear injection of a 10 kb *NotI-PacI* fragment containing pHb9-MitoEGFP. Five founders were identified via PCR, four of which were fertile, and lines were established and backcrossed into a C57Bl/6 background for at least three generations. Genotypes were determined by PCR using the following primers: 5′-CGTCAGGCTAATTGGACCGTG-3′, 5′-CCGTAGGTGGCATCGCCCTC-3′, and 5′-CTTACGGAGACTGAGCGTGGC-3′. Subline 34-116 Hb9-MitoEGFP mice were mated to previously described SOD1^G85R^ [14] and SOD1^G37R^ [13] mice. Resulting Hb9-MitoEGFP/SOD1 animals (or Hb9-MitoEGFP littermates) were perfused at various times in order to establish a time line for the disease in reference to mitochondrial shape and distribution.

### Immunoblotting, immunofluorescence and antibodies

Spinal cords were processed for immunoblot analysis as previously described [14] using a polyclonal anti-EGFP antibody and a monoclonal anti-α-tubulin (DM1A, Sigma) antibody.

Spinal cord, sciatic nerve, and L5 roots were dissected from animals transcardially perfused with phosphate-buffered 4% paraformaldehyde (FD NeuroTechnologies). Tissues were then post-fixed for 2 hours, cryoprotected, and then embedded in OCT (TissueTek) [14]. Sections (spinal cords: 30 µm; sciatic nerve and L5 roots: 10 µm) were labeled with one or more antibodies, including: SMI-32 (1∶2000, Sternberger Monoclonals), cytochrome c (1∶200, BD Biosciences), MBP (1∶200, Chemicon) or mAb A5C3 (misfolded SOD1, 1∶100; Gros-Louis et al., 2010). Labeling was visualized via fluorescently conjugated anti-mouse or rat antibodies (Cy5 or Texas Red; Jackson Immunochemicals). All antibodies were diluted in blocking buffer (3% fetal calf serum, 0.2% Tween-20 in PBS. Sciatic nerves were additionally labeled with Fluoromyelin Red (1∶300, Molecular Probes). All sections were mounted with ProLong Antifade reagent (Invitrogen) and analyzed with a confocal microscope (Leica SP5; 63x oil, 1.7 NA; digital zoom of 2–4.5x). A minimum of 2–5 animals per genotype for each age (each with 2–5 images each) were collected for analysis. For colocalization, Leica LAS AF software was used to calculate the overlap coefficient between misfolded SOD1 (A5C3) and individual mitochondria (MitoEGFP) in ventral roots. Specifically, MitoEGFP^+^ mitochondria were selected using the “region of interest” tool in 5–10 axons of each genotype (SOD1^G37R^: 10 axons with 34 mitochondria; SOD1^G85R^: 5 axons with 26 mitochondria) and the overlap coefficient was recorded. An overlap coefficient value of one represents the maximum degree of colocalization, and zero indicates no colocalization. This method has been applied previously [33].

### Quantitation of mitochondrial parameters and distribution

*Detection of mitochondria:* Image processing was performed using custom software written in MATLAB (MathWorks, Natick, MA). Confocal reconstructions were separated into monochromatic green and red channels, for detection and analysis of mitochondria (MitoEGFP) and axonal geometry (myelin), respectively. Blind deconvolution (four iterations, with an initial estimate for the size of the point spread function equal to four) was used to yield separation of individual mitochondria. Otsu's method was then used to generate a thresholded binary image. A series of morphometric operations was used to clear single pixels, break residual connections between mitochondria, and smooth the surface of each mitochondrion. These operations detected ∼90% of mitochondria accurately. The remaining 10% reflected mitochondria with more complex connecting linkages were separated manually, resulting in an overall detection error of <1% of mitochondria compared to manual selection.

#### Mitochondrial geometry, densities, and localization

Areas of each mitochondrion were calculated based on the number of pixels composing each separate object. Each mitochondrion was then fit to an elliptical shape, and the major and minor axes (mitochondrial length and width) as well as the coordinates of the ellipse (mitochondrial position) were determined. Aspect ratio was determined as the ratio of mitochondrial length to width; round mitochondria were represented by aspect ratios close to one.

Axonal geometry and coordinates for the center of SLIs were identified from the red (myelin) channel. Axons were divided into bins of 3.5 µm and mitochondrial density was calculated within each bin based on the coordinates of each mitochondrial centroid. Variability in the distribution of mitochondria along the length of the axon was quantified by fitting the histogram of mitochondrial density versus position along the axon with linear regression. Mitochondrial densities proximal to SLIs were calculated, respectively, based on the average mitochondrial density for a 7.0 µm region (two bins) proximal to each incisure. These were compared to mitochondrial densities for all remaining bins not associated with an incisure. Accumulation or clustering of mitochondria at SLIs was reflected by a ratio of SLI to non-SLI density greater than 1. Normal mitochondrial distribution along the length of the axon resulted in a ratio less than or equal to 1, based on the gradual tapering of axonal diameter at SLIs. Details of the number of mitochondria analyzed are in Table 1.

**Table 1:** Details on the number of individual mitochondria examined for all parameters.

	Cell Body	n	Axon	n
**Control**	4.5M	1987	4.5M	249
**G37R**		1250		101
**Control**	7M	1529	6M	526
**G85R**		2618		392
**Control**	8M	2115	8M	1043
**G85R**		3238		1841
**Control**	10M	630	10M	323
**G85R**		402		230

#### Statistics

Significant differences in mean values for parameters described above were tested using 2-way ANOVA analysis for time and genotype. ANOVA calculations for somal mitochondria area, length and aspect ratio were calculated as <0.0001. Axonal mitochondria length and aspect ratio also yielded ANOVA values <0.0001. Where data was significant for an interaction, we followed up with a post-hoc two-tailed t-test assuming unequal variances to assess differences between specific groups. t-test values are reported in text. Significant differences in distributions for parameters described above were tested using the Chi-square test with Yates' correction for continuity.
